# Comment on Eva *et al*. laparoscopic surgery: *Ann Med Surg (Lond)*. 2022 Sep 2;81:104562

**DOI:** 10.1097/MS9.0000000000000314

**Published:** 2023-03-14

**Authors:** Ospan A. Mynbaev, Nurlan S. Zhanabayev, Kaldybay S. Idrissov, Rauan Y. Botabayeva, Amirkhan K. Baimagambetov

**Affiliations:** a Moscow Institute of Physics and Technology National Research University, Dolgoprudny, Moscow Region, Russia; b New European Surgical Academy, Berlin, Germany; c Regional TB dispensary of the Health Department of Turkestan Region; d South Kazakhstan Multidisciplinary Medical College; e Department of General Practitioner, South Kazakhstan Medical Academy, Shymkent; f Department of Pharmaceutical Disciplines, Khoja Akhmet Yassawi International Kazakh-Turkish University, Turkistan, Kazakhstan; g Department of Specialized Surgical Disciplines, Khoja Akhmet Yassawi International Kazakh-Turkish University, Turkistan, Kazakhstan

*Dear Editor*,

Comment on Eva *et al*. Laparoscopic surgery: a randomized controlled trial comparing intraoperative hemodynamic parameters and arterial blood gas changes at two different pneumoperitoneal pressure values. *Ann Med Surg* (Lond). 2022 Sep 2;81:104562.

Recently, a randomized controlled trial comparing intraoperative hemodynamic parameters and arterial blood gas changes at two different pneumoperitoneum pressure regimens by Eva and colleagues was published in the *Annals of Medicine and Surgery* 1.

In this study, the authors demonstrated changes in hemodynamic, respiratory, blood gas, and acid–base parameters in two groups of patients during laparoscopic surgery with a CO_2_ pneumoperitoneum pressures of 8 and 12 mm Hg. Perioperative measurements were performed before and after anesthesia induction, 30 min after the start of the CO_2_ pneumoperitoneum, and 5 min after deflation^1^.

Since our research studies and clinical work have been closely related to the clinical and experimental aspects of CO_2_ pneumoperitoneum and its pathophysiology^2^–^5^, when we read this article, we found questionable results and shortcomings in this study.

From this view, unexpected respiratory rate and pH changes are bizarre and disputable in contrast to their observation concerning pCO_2_ dynamics during CO_2_ pneumoperitoneum 1. The pCO_2_ dynamics demonstrated increased carbon dioxide concentration during CO_2_ pneumoperitoneum with subsequently decreased pCO_2_ after deflation in both groups. However, the pH value in patients with CO_2_ pneumoperitoneum pressure of 12 mm Hg increased following the raised carbon dioxide concentration. Further, after deflation, decreased pCO_2_ was accompanied by increased pH in this group of patients with a CO_2_ pneumoperitoneum pressure of 12 mm Hg. These observations did not correspond to the physiologic balance between pCO_2_ and pH^1^.

When involved in laparoscopic surgery research, the prior knowledge for surgeons and anesthesiologists should be that pH is the general product of carbon dioxide. Dissolved carbon dioxide (CO_2_+H_2_O) in the human body forms a carbonic acid (H_2_CO_3_), which dissociates into two ions: hydrogen (H^+^) and bicarbonate (HCO_3_-). All side effects of CO_2_ pneumoperitoneum manifested in parameters of blood gases, acid–base balance, and hemodynamic system are triggered mainly by increased pCO_2_ with decreased pH and subsequent metabolic changes^4^.

It is well known that an accumulation of carbon dioxide in the body and its increased partial pressure (pCO_2_) are accompanied by a linearly decreased pH value. Such an observation was made in our previous experiments in ventilated rabbit models^5^. We demonstrated a constantly increased pCO_2_ value when the superficial ventilation mode remained unchanged. An increased pCO_2_ was accompanied by a decreased pH value overall during CO_2_ pneumoperitoneum. In animals with ventilated lungs without CO_2_ pneumoperitoneum, main blood gas, and acid–base parameters remained unchanged with slight events of alkalosis^3^,^4^.

Strangely, the respiratory rate increased enormously after deflation in patients with 12 mm Hg CO_2_ pneumoperitoneum pressure, although all disturbances of blood gas parameters in patients with 8 mm Hg pressure were in the frame of dynamic changes for these values during laparoscopic surgery and CO_2_ pneumoperitoneum with lung ventilation management 1.

The authors describe changes in blood gas and acid–base values in the results section needing clarification^1^. In addition, the CO_2_ pneumoperitoneum time is an essential factor with a crucial impact on blood gas and acid–base values, with subsequent disorders in cardiac and hemodynamic parameters. However, the authors did not describe the CO_2_ pneumoperitoneum time, types of surgeries, and Trendelenburg positioning during surgery^1^.

There is a physiological balance between carbon dioxide content and pH in the human body, which is usually regulated through respiratory system functioning and acid–base buffering systems of homeostasis. Therefore, during laparoscopic surgery with CO_2_ pneumoperitoneum, the minute volume is traditionally managed by the respiratory rate per minute and by the volume of inspiratory air per stroke (tidal volume) during lung ventilation. Subsequently, with the rise in carbon dioxide concentration, the minute volume is increased to keep the pCO_2_ value within the frame of the physiologic limits.

In experimental and clinical studies, we found that the intensity of increased carbon dioxide concentration and decreased pH values in the arterial blood were dependent on the CO_2_ pneumoperitoneum pressure level and lung ventilation accuracy during CO_2_ pneumoperitoneum^2^–^5^.

Another shortcoming of this study (Eva *et al*., 2022) is that sampling blood gas and acid–base parameters were not repeated during CO_2_ pneumoperitoneum and deflation. Blood gas and acid–base parameters are very sensitive to any confounding factors; therefore, sampling should be repeated to get the optimal values of these parameters.

Since this study aimed to compare arterial blood gas and hemodynamic changes between two CO_2_ pneumoperitoneum pressures, the authors should estimate the CO_2_ pneumoperitoneum time. Considering fewer cases in groups, there might be highly distributed blood gases and acid–base values.

In addition, the authors of their randomized controlled trial should have used the correct study design and statistics^1^. This study had 10 patients per group; a suitable study design should be used with nonparametric statistical tests.

We expect an explanation from the authors for such an unexpected observation of pH and respiratory rate distributions when the dynamics of these parameters should be in the frame of limits according to changes in the carbon dioxide partial pressure monitored during surgery.

## Ethical approval

NA.

## Consent

NA.

## Sources of funding

None.

## Author contribution

All authors contributed to conceptualization, data curation, investigation, methodology, and writing.

## Conflicts of interest disclosure

Nothing to disclose.

## Research registration unique identifying number (UIN)

1. Name of the registry: NA.
2. Unique identifying number or registration ID: NA.
3. Hyperlink to your specific registration (must be publicly accessible and will be checked): NA.

## Guarantor

Ospan A Mynbaev.

## Provenance and peer review

Not commissioned, externally peer reviewed.

## References

[1] Eva I Rosario V Guglielmo R . Laparoscopic surgery: a randomized controlled trial comparing intraoperative hemodynamic parameters and arterial blood gas changes at two different pneumoperitoneal pressure values. Ann Med Surg (Lond). 2022;81:104562. doi:10.1016/j.amsu.2022.104562. 36147166 PMC9486856

[2] Ormantayev AK Sepbayeva AD Kosmas IP . Comment on ‘evidence for negative effects of elevated intra-abdominal pressure on pulmonary mechanics and oxidative stress’. ScientificWorldJournal 2015;2015:746937. 26171420 10.1155/2015/746937 PMC4480807

[3] Mynbaev OA Eliseeva MY Kalzhanov ZR . Surgical trauma and CO_2_-insufflation impact on adhesion formation in parietal and visceral peritoneal lesions. Int J Clin Exp Med 2013;6:153–165. 23573346 PMC3609691

[4] Mynbaev OA Molinas CR Adamyan LV . Reduction of CO_2_-pneumoperitoneum-induced metabolic hypoxaemia by the addition of small amounts of O_2_ to the CO_2_ in a rabbit ventilated model. A preliminary study. Hum Reprod 2002;17:1623–1629. 12042288 10.1093/humrep/17.6.1623

[5] Mynbaev OA Molinas CR Adamyan LV . Pathogenesis of CO_2_ pneumoperitoneum-induced metabolic hypoxemia in a rabbit model. J Am Assoc Gynecol Laparosc 2002;9:306–314. 12101327 10.1016/s1074-3804(05)60409-4

